# The Quality of Greek Oregano (*O*. *vulgare* L. subsp. *hirtum* (Link) Ietswaart) and Common Oregano (*O*. *vulgare* L. subsp. *vulgare*) Cultivated in the Temperate Climate of Central Europe

**DOI:** 10.3390/foods9111671

**Published:** 2020-11-15

**Authors:** Zenon Węglarz, Olga Kosakowska, Jarosław. L. Przybył, Ewelina Pióro-Jabrucka, Katarzyna Bączek

**Affiliations:** Department of Vegetable and Medicinal Plants, Institute of Horticultural Sciences, Warsaw University of Life Sciences–SGGW, 02-787 Warsaw, Poland; zenon_weglarz@sggw.edu.pl (Z.W.); jaroslaw_przybyl@sggw.edu.pl (J.L.P.); ewelina_pioro_jabrucka@sggw.edu.pl (E.P.-J.); katarzyna_baczek@sggw.edu.pl (K.B.)

**Keywords:** *Origanum* subspecies, morphological traits, glandular trichomes, essential oil composition, rosmarinic acid, sensory evaluation

## Abstract

The purpose of the study was to determine the differences between two subspecies: *O*. *vulgare* L. subsp. *hirtum* (Link) Ietswaart (Greek oregano) and *O*. *vulgare* L. subsp. *vulgare* (common oregano) growing in cultivation conditions within temperate climate of Central Europe. The characteristic of the subspecies was undertaken in terms of selected morphological parameters and the quality of the raw material. The herb of both subspecies was evaluated on the content and composition of essential oil by hydrodistillation followed by GC-MS and GC-FID (gas chromatography coupled with mass spectrometry and flame ionization detector), the total content of phenolic acids (according to PP 6th ed.) and the content of rosmarinic acid (by HPLC). The sensory evaluation (QDA) was performed, as well. Greek oregano was distinguished by visibly higher number of glandular trichomes on the leaves (up to 4.85 per 1 mm^2^) followed by higher content of essential oil in the herb (up to 3.36 g × 100 g^−1^ DW) in comparison to common oregano. Based on the essential oil composition, Greek oregano was classified as mixed carvacrol/γ-terpinene chemotype, while common oregano as mixed sabinyl/cymyl type rich in sesquiterpenes. Greek oregano was also characterized by higher total content of phenolic acids (up to 6.16 g × 100 g^−1^ DW) and rosmarinic acid (up to 6787.2 mg × 100 g^−1^ DW) than common oregano. Essential oil content reached the maximum at the beginning of blooming (common oregano) and at the full blooming stage (Greek oregano). In turn, the amount of phenolic acids followed by rosmarinic acid was the highest at the beginning of seed-setting stage, in the case of both subspecies. The differences between subspecies concerning chemical composition (especially essential oil) were reflected in the sensory attributes, where both odor and taste notes were found at higher level for Greek oregano. Results of our work indicate that Greek oregano is well adapted to grow in the temperate zone conditions. Such adaptation was reflected mainly in the satisfied yield and maintaining characters typical for the Mediterranean plant, e.g., a high essential oil content followed by high carvacrol share, traits the most important from practice viewpoint.

## 1. Introduction

Plants belonging to *Origanum* genus (*Lamiaceae* family) have been known as culinary and medicinal plants since ancient times. This genus contains 49 taxa belonging to 10 sections. Some species, including *Origanum vulgare* L., are rich in essential oil and commonly known as “oregano” [1]. *Origanum vulgare* L., an aromatic, perennial sub-shrub, is widely distributed all over Eurasia and North Africa [2]. The species is regarded to be extremely variable, both in its morphological features and chemical composition. Given its specific biological character and significant economic importance, *O*. *vulgare* has been placed in the List of Priority Species in Europe [3]. According to the widely accepted taxonomy, six subspecies of *O*. *vulgare* have been recognized [2,4]. Among them, *O*. *vulgare* L. subsp. *hirtum* (Link) Ietswaart so-called Greek oregano, endemic to the Mediterranean area, is cultivated almost all over the world and regarded as the most valuable one [5]. Another subspecies important from economic point of view, is *Origanum vulgare* L. subsp. *vulgare* (common oregano). It frequently occurs on the region of Northern and Central Europe and is the only representative of *O*. *vulgare* in Poland [6,7]. The upper, not woody parts of flowering shoots (herb) of both subspecies is commonly used and traded raw material. Besides the range of occurrence, these two subspecies differ in terms of many features, whereas the content and composition of essential oil seems to be the most important, because it determines medicinal properties of the herb and its sensory value [2]. Greek oregano is rich in essential oil (about 5%), while common oregano contains less amount (up to 2%) of this substance. Subspecies create few various chemotypes defined on the basis of the dominant compound in essential oil. Greek oregano accumulates mainly phenolic monoterpenes (thymol and carvacrol) followed by its precursors (*p*-cymene and γ-terpinene). In turn, common oregano is distinguished by less active biosynthesis of “cymyl” compounds in favor of the bicyclic “sabinyl” (i.a. sabinene, *cis*/*trans* sabinene hydrate and its acetates) or acyclic once (i.a., β-ocimene, β-myrcen, linalyl acetate, linalool). This kind of chemotype is often accompanied by high content of sesquiterpenes (i.a. germacrene D, β-caryophyllene and caryophyllene oxide) [1,7,8,9,10,11,12,13,14,15,16,17,18]. Both *Origanum* subspecies contain also considerable amounts of non-volatile phenolic compounds such as flavonoids and phenolic acids. Rosmarinic acid followed by caffeic, vanillic, *o*-coumaric and protocatechuic acids dominate in common oregano herb [7,19,20]. When given Greek oregano, rosmarinic and lithospermic acids are the present in the highest amounts [13,21,22]. In both subspecies, flavonoids are represented mainly by derivatives of luteoline and apigenine [7,13,20,21,22,23,24]. In relation with such a wide range of biologically active compounds, both *Origanum* subspecies indicate various pharmacological activities, especially antimicrobial, choleretic and antioxidant. Common oregano herb reveals also diuretic and expectorant properties, while Greek oregano—stimulative, carminative, antispasmodic, and anticancer [25,26]. It is worth noting that Greek oregano is listed in the European Pharmacopeia and is recommended as a remedy for gastrointestinal disorders treatment, temporary loss of appetite and to stimulate bile secretion [25,26,27]. Common oregano, even though not mentioned in European Pharmacopeia, used to be applied in the same way in both modern and folk medicine [28]. Both *Origanum* subspecies are widely used not only in pharmaceutical industry but also as a food preservative and flavoring, cosmetic ingredient, and, most importantly—as a culinary herb [29,30].

Despite abovementioned intraspecific diversity, *O*. *vulgare* is still treated as a collective taxon [31,32,33,34]. Moreover, many varieties, landraces, forms, ecotypes, and cultivars are nowadays available for stakeholders, creating possibility of subspecies misleading [35]. This altogether may lead to decrease homogeneity and the quality of raw material. It is especially important, since herbal products standardization requirements are taken into consideration [36].

Up to now, Greek oregano cultivation areas have been located mainly within a warmer climate. Recent studies have showed that this subspecies may be cultivated in temperate zone of Central Europe, as well [13,37,38]. However, in Poland, the cultivation of this subspecies is at its infancy [18]. In turn, common oregano used to be collected in Poland both from natural sites and cultivation [28]. However, the harvest from the wild may result in heterogeneous raw material. Moreover, the number of wild growing common oregano populations has recently significantly decreased what can lead to genetic erosion. Thus, the collection of common oregano herb exclusively from cultivation would provide natural resources protection as well as ensure high quality of raw material [39].

The aim of the study was to determine the differences between Greek oregano and common oregano in cultivation conditions within temperate climate of Central Europe. The characteristic of the subspecies was undertaken in terms of selected morphological parameters and the quality of raw material, reflected in the content of biologically active compounds (the total content and composition of essential oil and phenolic acids) and sensory evaluation.

## 2. Materials and Methods

### 2.1. Plant Material

The experiment was carried out at the experimental field of the Department of Vegetable and Medicinal Plants, Warsaw University of Life Sciences (WULS-SGGW) (5210180 N; 2105234 E), on heavy alluvial soil. Seeds of Greek oregano and common oregano originated from Polish Gene Bank collection (accession numbers: 406735 and 401291, respectively). Seeds were sown in the first week of February (2020) into multi-pots filled with a peat substrate, in a greenhouse. A total of 180 seedlings of each subspecies were randomly selected and planted out into the field in the last week of April. The randomized block design (60 seedlings per plot; in 3 replications) was applied, with a spacing of 40 × 60 cm. The harvest of the herb (upper, not woody parts of shoots) was performed on 1-year old plants, at three stages of plant’s development: at the beginning of blooming (fourth week of June), at the full blooming (third week of July) and at the beginning of seed-setting (second week of August). The herb was cut at a height of about 15 cm above ground. The fresh and dry weight of the herb was determined (g per plant). After drying at 35 °C, the herb was ground and prepared for chemical analysis. Climatic parameters were recorded (Table 1).

### 2.2. Morphological Observations

**Figure 1:**
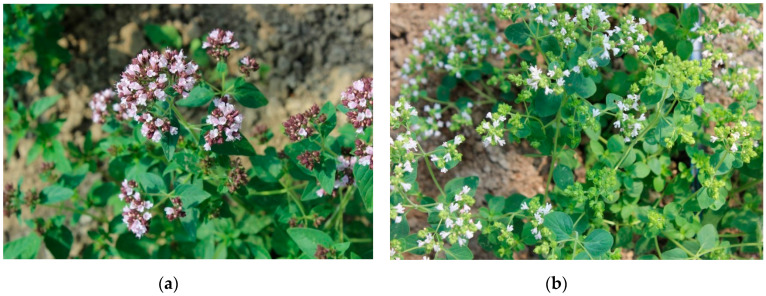
Common oregano (**a**) and Greek oregano (**b**).

**Figure 2:**
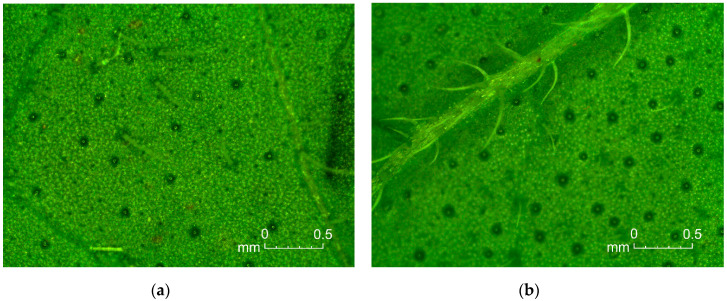
Glandular trichomes on abaxial leaf surface of common oregano (**a**) and Greek oregano (**b**).

Morphological characters were evaluated according to the List of Descriptors for *Origanum vulgare* L. elaborated by the Medicinal and Aromatic Plants Working Group of European Cooperative Programme for Plant Genetic Resources (MAPs WG ECP/GR) [40]. Observations were carried out directly before the first harvest of raw material, on 10 plants per subspecies. Following traits were determined: plant growth habit, plant height (cm), number of shoots per plant, number of internodes per shoots, color of petals, branching density, stem pubescence, color of stem, degree of lignification, foliage density, shape of leaf blade, leaf area, leaf margin and shape of leaf apex. Moreover, microscopic observations concerning density of glandular trichomes on abaxial and adaxial surface of the leaves were evaluated, according to the method described by Kosakowska et al. [41]. Photographic documentation was performed (Figure 1 and Figure 2).

### 2.3. Chemical Analysis

#### 2.3.1. Content of Essential Oil

A total of 50 g of air-dried herb was subjected for hydrodistillation for 3 h using a Clevenger-type apparatus. The content of essential oil was expressed as g × 100 g^−1^ of dry weight (DW). Essential oils were collected and stored in amber vials, at 4 °C.

#### 2.3.2. Analysis of Essential Oils by GC-MS and GC-FID (Gas Chromatography Coupled with Mass Spectrometry and Flame Ionization Detector)

The analysis was carried out by usage of an Agilent Technologies 7890A gas chromatograph coupled with a flame ionization detector (FID) and MS Agilent Technologies 5975C Inert XL_MSD with Triple Axis Detector (Agilent Technologies, Wilmington, DE, USA). Polar, capillary, HP 20M column (25 m × 0.32 mm × 0.3 µm film thickness) (Agilent Technologies, Wilmington, DE, USA) was used. Separation conditions were given previously by Bączek et al. [42].

#### 2.3.3. Total Content of Phenolic Acids

The analyses (Arnov’s method) was performed in accordance with Polish Pharmacopeia 6th ed. [43]. A total of 1 g of air-dry, grounded herb was extracted twice with portions of 25 mL of distilled water (a total of 50 mL), with shaking for 30 min each time at room temperature (a total of 1 h). Collected extract was filled to 50 mL with distilled water. A total of 1 mL of extract was mixed with 5 mL of distilled water, 1 mL 0.5 M HCl, 1 mL of Arnov reagent (10 g of sodium molybdate and 10 g of sodium nitrite dissolved in 100 mL of distilled water) and 1 mL 1 M NaOH and subsequently completed to 10 mL with distilled water. The absorbance of both basic (with extract) and comparison (without extract) solutions were measured at 490 nm. The total phenolic acid content was recalculated and given as caffeic acid equivalent (g × 100 g^−1^ DW).

#### 2.3.4. Analysis of Phenolic Acids by HPLC-DAD (High Pressure Liquid Chromatography Coupled with Diode Array Detector) 

The sample preparation, parameters of chromatographic separation and integration as well as validation procedure was given earlier by Kosakowska et al. [41]. The content of rosmarinic acid was calculated in mg × 100 g^−1^ DW.

### 2.4. Sensory Analysis

Sensory evaluation was carried out in the sensory laboratory of the Department of Vegetables and Medicinal Plants, WULS-SGGW. Quantitative descriptive analysis (QDA) was used. The evaluation was determined on the fresh herb of both subspecies, collected in the first cut (at the beginning of blooming). Attributes of its taste and odor were selected and estimated. In order to select attributes, ‘brainstorming’ sessions were done by an expert panel consisting of a minimum of 10 assessors. Evaluation was performed in two independent sessions. The description of method has already been given by Kosakowska et al. [18].

### 2.5. Statistical Analysis

Data were subjected to statistical analysis using Statistica 12 software (Cracov, Poland). The mean values were compared by using the one way analysis of variance (ANOVA) followed by Tukey’s multiple range test. The differences between individual means were deemed to be significant at *p* < 0.05. Standard deviation (±SD) was estimated.

## 3. Results and Discussion

Investigated subspecies differed in both morphological and chemical traits (Table 2, Table 3, Table 4, Table 5 and Table 6). Common oregano was characterized by erect type of growth and ligneous, slightly hairy stems. The color of stems was dark green and red, while petals were pink. The plant height was at a level of 36.11 cm. In turn, Greek oregano was distinguished by semi-erect type of growth, and green, slightly ligneous but hairy stems. This subspecies was characterized by white color of petals. Greek oregano plants grown in Poland were about 10 cm lower than common oregano plants. The foliage density was described as medium (in common oregano) and dense (in Greek oregano). The branching density was sparse in both subspecies. Number of shoots per plant achieved values 27.59 in the case of common oregano, and 22.77 in Greek oregano. The number of internodes per shoot was similar in both subspecies (8.44; 7.60, respectively) (Table 2 and Table 3). Obtained results indicate on significant differences between examined *Origanum* subspecies and correspond well with the literature data [14,17,19,44,45,46,47,48,49]. However, it should be underlined that each subspecies is very variable itself and its morphological features strongly depend on the population/accession origin. For instance, common oregano plant’s height ranged from 18 to 59 cm [46], while Greek oregano—from 67.8 to 79.9 cm [49]. Observed phenotypical plasticity may be related to allogamous way of this plant’s reproduction as well as its heterozygous character. Traits such as type of growth habit, lignification degree as well as branching and foliar density can be important from the practical viewpoint, since they affect the yield of herb and enable its mechanical harvest [17]. In the present study, the fresh and dry weight of common oregano herb was slightly higher (63.81; 16.71 g × plant^−1^) in comparison to Greek oregano (49.17; 13.28 g × plant^−1^) (Table 3). Such results may be related to high temperature requirements of Greek oregano resulting from its Mediterranean origin. Taking into consideration the possible response of this subspecies to climatic parameters, its cultivation under covers may be effective. Results obtained by Kosakowska et al. [18] showed that Greek oregano plants cultivated under foil were distinguished by almost twice the mass of the herb when compared to those grown without covers. In general, Greek oregano cultivation is widely presented in literature, however the majority of these data concerns warm climate zones [50,51,52,53].

Examined *Origanum* subspecies cultivated in Poland varied also in terms of leaves parameters. Leaves of common oregano were characterized by higher area of blade than Greek oregano (78.27 and 61.76 mm^2^, respectively) (Table 4). Moreover, they were distinguished by ovate shape and acute apex, while in the case of Greek oregano, the shape of leaf blade was rhomboid with rounded apex. Leaves of both subspecies had denticulate leaf margin (Table 2). Another feature that strongly differentiated common oregano and Greek oregano leaves was the density of glandular trichomes situated on the upper and down leaf surface (Table 4). In *Origanum* subspecies (as well as in other Lamiaceae), glandular trichomes are multicellular epidermal glands responsible for storage of essential oil. Two different types of these glands were recognized on the epidermis of *Origanum* species: peltate and capitate glands. The glandular trichomes are built of one basal cell, one stalk cell and a multi-cellular head, where essential oil is synthesized before being transferred to subcuticular area [54,55,56]. Svidenko et al. [56] claim that the location of glandular trichomes have valuable taxonomical significance at the species level. In the present work, the number of glandular trichomes per 1 mm^2^ was significantly higher when given Greek oregano leaves (4.78 on adaxial and 4.85 on abaxial surface) in comparison to common oregano (0.78 and 1.17, respectively) (Table 4). This pattern corresponds with studies undertaken earlier by Shafiee-Hajiabad et al. [57]. However, the author showed higher number of glands in both subspecies: up to 17 per 1 mm^2^ in Greek oregano and up to 9.67 per 1 mm^2^ in common oregano. This inaccuracy may be related to the phenomenon that the formation of glandular trichomes is variable and can be controlled by both genetic and environmental factors [54,58].

When given aromatic plants, including oregano, the problem concerning the content and composition of essential oil seems to be one of the most important, because this substance is responsible for both sensory value and pharmacological activity of the raw material. In the present work, in the case of common oregano, the essential oil content ranged from 0.27 to 0.49 g × 100 g^−1^ DW, with the maximum noticed at the beginning of plant’s blooming. In turn, in Greek oregano the amount of this substance varied from 2.75 g × 100 g^−1^ DW (beginning of blooming) to 3.36 (full blooming stage) (Table 5). These results support the thesis that common oregano belongs to essential oil-poor group of *Origanum* subspecies, while Greek oregano represents the essential oil-rich group [2]. It is worth noting that the relationship between the number of glandular trichomes and essential oil content has been found (Table 4 and Table 5), what refers to results shown by Shafiee-Hajiabad et al. [57]. Moreover, obtained results correspond with the phenomenon that the oregano essential oil fluctuates during vegetation season and usually reaches the maximum level at the full blooming stage of plant’s development, therefore this time used to be regarded as the best for harvest [13,38]. It is known that many various factors can affect the content and composition of essential oils in aromatic plants, where the most seem to be: genetic, physiological and environmental including temperature, intensity of solar and radiation humidity [59,60,61].

In the present study, 25 compounds were identified in the common oregano essential oil, forming up to 98.11% of total identified fraction. In the case of Greek oregano, 24 constituents were detected, accounting up to 98.89%. The monoterpenes created the fundamental part in both essential oils, with a domination of monoterpene hydrocarbons comprising up to 53.43% and 53.27%, respectively. In Greek oregano, phenolic monoterpenes were also present in the considerable amounts (up to 32.75%). Carvacrol took the majority of this fraction (up to 32.02%), while monoterpene hydrocarbons part was formed mainly by γ-terpinene (up to 28.00%). The domination of above listed compounds let to qualify investigated Greek oregano essential oil as mixed carvacrol/γ-terpinene chemotype. According to literature data, this subspecies is able to create various chemotypes (pure or mixed), based on the dominant compound, such as: carvacrol, tymol, *p*-cymene and γ-terpinene [8,18,38,57]. In present work it was observed that the percentage share of carvarol in Greek oregano essential oil increased from the beginning of blooming to the beginning of the seed-setting stage of plant’s development (28.35, 32.02% respectively), in parallel with γ-terpinene decrease (from 28.00 to 19.62%) (Table 5). These results agree with those shown by Grevsen et al. [13] and correspond to Hudaib et al. [62] studies, indicating that phenolic monoterpenes (thymol and carvacrol) and their precursors (γ-terpinene and *p*-cymene) show synchronized patterns of variations during vegetation season. Taking into consideration that the synthesis of monoterpenes can be affected by temperature, obtained results may be related to the plant’s physiological response for this climatic parameter [61].

The results of our work indicate on the domination of sabinene in common oregano essential oil. This compound represents monoterpene hydrocarbons fraction. Its content was at the similar level during plant’s vegetation: 27.16% at the beginning of blooming, 27.60% at the full blooming and 26.42%—at the beginning of seed-setting. Sabinene was accompanied by other monoterpenes present in amounts not exceeding 10%, i.a.: *p*-cymene, 1.8 cyneol, linalool, terpinolene, etc. Interestingly, there was also a high content of phenolic monoterpenes (carvacrol and thymol) in analyzed common oregano samples (up to 15.89 and 3.57%, respectively). Besides monoterpens, the sesquiterpenes fraction was found in considerable amounts, with β-caryophyllene and its oxide as dominants (Table 5). Thus, such a chemical composition allows to classify this essential oil as mixed sabinyl/cymyl type rich in sesquiterpenes. Sabinyl chemotypes are regarded to be the most frequent within common oregano subspecies, while the occurrence of phenolic monoterpenes is rather rare [7,16]. Based on the literature data, it seems that common oregano is more polymorphic than Greek oregano, since a lot of different chemotypes have been distinguished, as following: *p*-cymene + β-caryophyllene, germacrene D + β-caryophyllene, sabinene, cis-sabinene hydrate, terpinen 4-ol, etc. [9,10,11,17,44]. Irrespectively of the subspecies, carvacrol or/and thymol chemotypes are considered to be the most valuable in the view of medicinal activities (especially antimicrobial) of these phenolic monoterpenes [25]. Moreover, these substances are responsible for sensory properties of the raw material, in particular: its herbal and spicy aroma [63,64]. According to European Pharmacopeia 9th, the sum of thymol and carvacrol in Greek oregano should not be lower than 60% [27]. Thus, phenolic chemotypes seem to be interesting for industrial purposes, especially pharmaceutical and food. Obtained results indicate that investigated Greek oregano accession doesn’t meet EP requirements. However, acyclic (e.g., rich in linalool) or sesquiterpenes (e.g., rich in β-caryophyllene) as well as sabinyl chemotypes can be valuable from practical point of view, as well. For instance, due to pleasant floral aroma of linalool, chemotypes rich in this constituent (occurring in common oregano) may be used in cosmetic and perfumery industry [65].

**Table 5:** The total content (g × 100 g^−1^ DW) and gas chromatographic composition (% peak area) of essential oil samples.

				Common Oregano *O. vulgare* ssp. *vulgare*			Greek Oregano *O. vulgare* ssp. *hirtum*		
No	Compound	RI^a^	RI^b^	Beginning of Blooming	Full Blooming	Beginning of Seed-Setting	Beginning of Blooming	Full Blooming	Beginning of Seed-Setting
1	α-thujene	1023	1012–1039	1.30	1.85	1.46	4.11	4.39	1.73
2	α-pinene	1028	1008–1039	0.44	0.57	0.42	0.29	0.28	2.45
3	camphene	1076	1043–1086	0.03	0.04	0.05	0.78	0.80	2.04
4	β-pinene	1113	1085–1130	2.58	2.57	1.73	4.01	3.23	3.20
5	sabinene	1125	1098–1140	27.16	27.60	26.42	0.12	0.51	2.61
6	3-carene	1145	1122–1169	0.00	0.00	0.00	0.17	0.16	0.06
7	α-terpinene	1183	1154–1195	1.01	1.53	1.29	5.30	4.28	3.43
8	D-limonene	1206	1178–1219	0.87	1.10	0.75	0.33	0.32	3.72
9	α-phellandrene	1210	1148–1186	0.00	0.00	0.00	0.43	0.41	0.88
10	1.8 cyneol	1213	1186–1231	3.62	3.34	2.66	0.00	0.00	0.00
11	*trans* β-ocimene	1235	1211–1251	0.77	1.48	1.44	0.09	0.13	0.10
12	γ-terpinene	1248	1222–1266	2.46	4.20	3.51	28.00	22.99	19.62
13	*p*-cymene	1273	1246–1291	6.85	8.53	6.29	8.88	14.53	9.13
14	*m*-cymene	1277	1244–1279	0.41	0.62	0.43	0.00	0.00	0.00
15	terpinolene	1284	1261–1300	3.62	3.34	2.66	0.34	1.24	0.00
16	1-octen-3-ol	1445	1411–1465	1.98	2.62	2.33	1.01	0.43	1.73
17	linalool	1542	1507–1564	4.06	4.78	3.84	0.75	3.45	0.86
18	β-caryophyllene	1596	1570–1685	7.84	8.19	8.49	2.80	1.18	2.70
19	terpinen-4-ol	1597	1564–1630	4.04	5.09	3.22	3.68	2.42	3.13
20	*cis*-terpineol	1620	1616–1644	0.20	0.39	0.69	0.80	0.22	0.75
21	*trans*-terpineol	1670	-	0.21	0.15	0.36	0.30	0.85	0.30
22	borneol	1684	1653–1728	0.00	0.05	0.00	2.86	2.74	3.02
23	β-bisabolene	1741	1698–1748	0.00	0.00	0.00	1.90	2.54	2.19
24	β-ionone	1845	1892–1958	0.21	0.15	0.07	0.00	0.00	0.00
25	caryophyllene oxide	1976	1936–2023	9.95	9.04	10.19	0.20	0.09	0.44
26	humulene oxide II	2017	1992–2083	1.19	0.67	0.71	0.00	0.00	0.00
27	thymol	2165	2100–2205	3.57	2.47	2.40	0.79	0.83	0.73
28	carvacrol	2214	2140–2246	10.63	5.68	15.89	28.35	30.87	32.02
29	α-cadinol	2228	2180–2255	1.14	1.06	0.81	0.00	0.00	0.00
	Total identified			94.84	97.11	98.11	96.29	98.89	96.84
	Monoterpene hydrocarbons			47.5	53.43	46.45	52.85	53.27	48.97
	Oxygenated monoterpenes			12.34	13.95	10.84	8.39	9.68	8.06
	Phenolic monoterpenes			14.2	8.15	18.29	29.14	31.7	32.75
	Sesquiterpene hydrocarbons			7.84	8.19	8.49	4.7	3.72	4.89
	Oxygenated sesquiterpenes			12.28	10.77	11.71	0.2	0.09	0.44
	Other compounds			1.98	2.62	2.33	1.01	0.43	1.73
	Essential oil content			0.49	0.27	0.40	2.75	3.36	3.10

RI^a^—experimental retention index on polar HP 20M column, RI^b^—range of retention indexes on polar column reported by Babushok et al. [66].

Another group of metabolites conditioning medicinal and sensory value of oregano herb are phenolics. Phenolic acids and flavonoids reveal various pharmacological activities as well as contribute to the color and flavor profile of plants [67,68]. Within phenolic acids, rosmarinic acid is a dominant compound in the Lamiaceae species, including *O. vulgare* [7,20,22,23,69]. This acid belongs to cinnamic acids derivatives. It is a depside, built on the basis of caffeic and 3, 4-dihydroxyphenyl lactic acids. Taking into consideration its extremely high antioxidant and antimicrobial activity, it may be used as a raw material quality marker [70,71]. In the present work, the content of rosmarinic acid in common oregano herb ranged from 2370.0 to 4998.9 mg × 100 g^−1^ DW, while in Greek oregano from 4569.0 to 6787.2 mg × 100 g^−1^ DW (Table 6). In both subspecies, the consequent increase of this compound (from the beginning of blooming until the beginning of the seed-setting phase) was noticed. Interestingly, a similar pattern was observed in the case of phenolic acids total content (2.65–4.89 and 4.63–6.16 g × 100 g^−1^ DW, respectively) (Table 6). Such phenomenon can be associated with the physiological function of these metabolites, which as natural antioxidants, are generally involved in mechanisms of plant protection and defense [72]. Moreover, as lignin’s components, phenolic acids make cell walls stronger [73]. Thus, plants being at the beginning of the seed-setting period may be more resistant to various stresses, than the younger ones, what is reflected in higher phenolic acids content.

**Table 6:** The total content of phenolic acids (g × 100 g^−1^ DW) and rosmarinic acid content (mg × 100 g^−1^ DW).

	Common Oregano *O. vulgare* ssp. *vulgare*			Greek Oregano *O.vulgare* ssp. *hirtum*		
Compound	Beginning of Blooming	Full Blooming	Beginning of Seed-Setting	Beginning of Blooming	Full Blooming	Beginning of Seed-Setting
Total content	2.65 ± 0.28 a	2.52 ± 0.26 a	4.89 ± 0.83 b	4.63 ± 0.52 A	4.97 ± 0.42 A	6.16 ± 0.30 B
Rosmarinic acid	2370.0 ± 258.6 a	4762.3 ± 415.0 b	4998.9 ± 263.0 b	4569.0 ± 249.5 A	4992.5 ± 301.5 A	6787.2 ± 608.4 B

Values marked in rows with different letters differ at *p* < 0.05.

**Figure 3:**
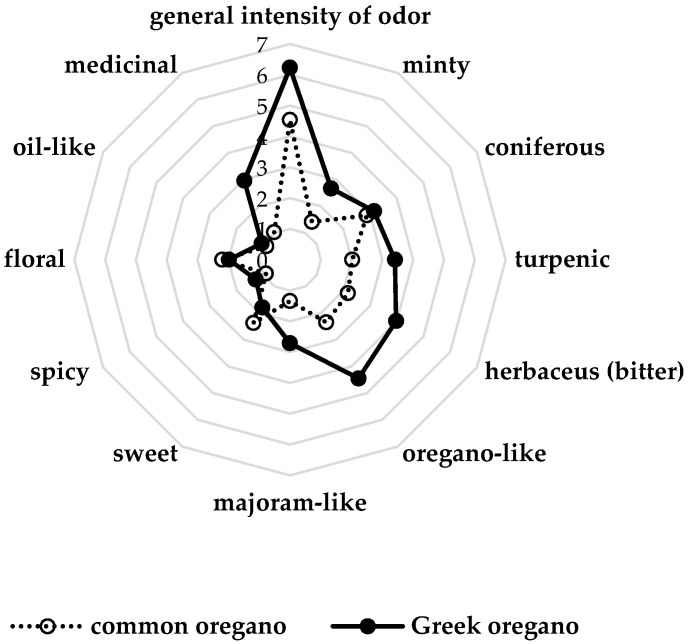
Sensory profile of herb odor of common oregano and Greek oregano.

**Figure 4:**
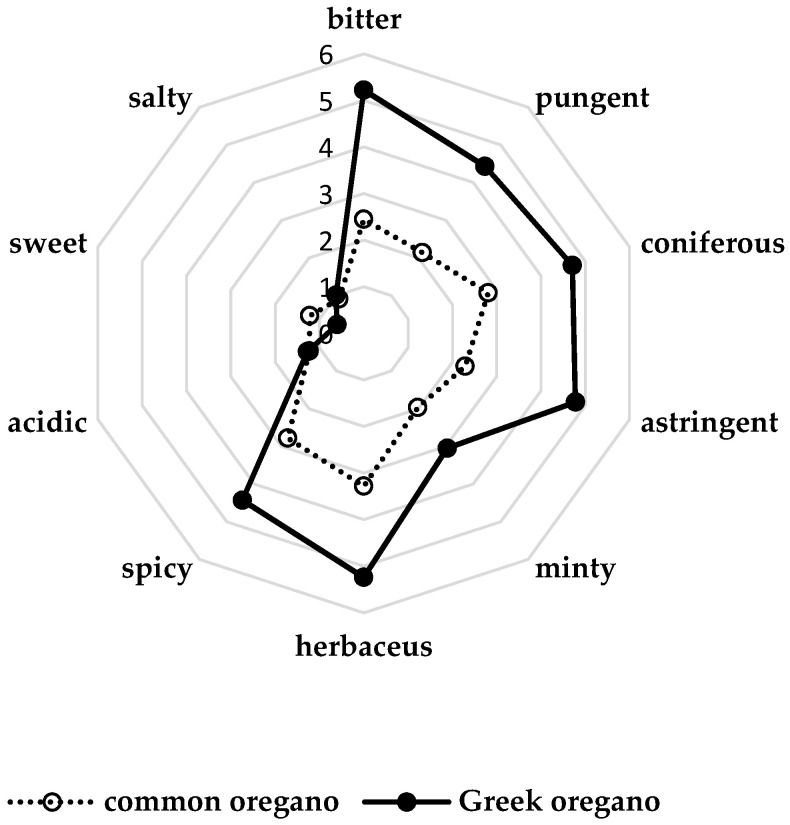
Sensory profile of herb taste of common oregano and Greek oregano.

In the case of culinary herbs, the organoleptic characteristic and their acceptance by consumers are important issues. Unpleasant flavor may be a reason of the rejection of the product, even though its quality meets Pharmacopeia or ISO (International Organization for Standardization) specifications [74]. Therefore, the sensory evaluation seems to be a crucial factor affecting the overall quality of spices. Results of sensory analysis, carried out in the present work, indicate on visible differences between odor and taste attributes of common oregano and Greek oregano (Figure 3 and Figure 4). Following notes were selected for odor: minty, coniferous, turpenic, herbaceous (bitter), oregano-like, majoram-like, sweet, spicy, floral, oil-like and medicinal. General intensity of odor was estimated, as well. When given taste: bitter, pungent, coniferous, astringent, minty, herbaceous, spicy, acidic, sweet and salty attributes were chosen. With regards to odor, it was observed that notes of Greek oregano herb were higher in comparison to common oregano, expect from sweet and floral ones (Figure 3). Similarly, when taste attributes were concerned: they were noticed at higher level for Greek oregano herb, apart from the sweet note. However, acidic and salty taste was described at the similar level for herb of both examined subspecies (Figure 4). In the case of *Origanum* plants, the sensory profile is conditioned mainly by its essential oil content and composition. As it was mentioned before, in our work, common oregano was qualified as mixed sabinyl/cymyl type rich in sesquiterpenes, while Greek oregano as mixed carvacrol/γ-terpinene chemotype (Table 5). Here, the more intense odor and taste of Greek oregano was probably related to carvacrol domination in its essential oil. Sensory attributes of carvacrol are defined as spicy, herbal, medicinal, phenolic, woody, cedar and pungent [64]. Other volatiles present in Greek oregano essential oil in the considerable amounts, such as γ-terpinene and *p*-cymene, also may affect its sensory profile. Odor of both substances is regarded as gasoline and citrus, while γ-terpinene is additionally described as herbaceous and turpentine [75]. The results obtained in our previous work showed that sensory profile of Greek oregano may be affected by the cultivation method [18]. According to Bonfanti et al. [29] and Asensio et al. [76], it may be related with methods of raw material conservation, as well.

## 4. Conclusions

Results obtained in the present work indicate on quite good adaptation of Greek oregano to climatic conditions of Central Europe. This subspecies, grown in the temperate zone, is able to create satisfied yield and still keeps its typical characters of the Mediterranean plant. Among them, a high amount of essential oil followed by a high percentage share of carvacrol seem to be the most important from the practice point of view. Common oregano also presented interesting features, especially when its chemotype (sabinyl/cymyl type rich in sesquiterpenes) and sensory value (floral, sweet) are concerned. Herb of both subspecies appeared to be a rich source of rosmarinic acid, a compound known for its extremely high antioxidant properties. It was shown that the content of this substance fluctuated during plant’s development: increased from the beginning of blooming to the beginning of seed-setting, both in Greek oregano and common oregano. With regards to the obtained results, it seems that Greek oregano can be successfully cultivated in the temperate climate of Central Europe. The production of this herb on site, which usually results in its lower price, may increase its availability and utilization, not only as a spice but also as natural medicine.

## Figures and Tables

**Table 1:** Climatic parameters in the vegetation season of 2020.

Months	Temperature (°C)	Rainfall (mm)	Air Humidity (%)	Sun Hours	Sun Days
April	12	13.1	53	210	17
May	15	127.1	64	157.5	4
June	22	108.2	68	179	3
July	22	43.2	61	225	6
August	24	70.1	62	295	12

**Table 2:** Morphological traits of investigated plants.

	Common Oregano *O. vulgare* ssp. *vulgare*	Greek Oregano *O. vulgare* ssp. *hirtum*
Plant habit	erect	semi-erect
Color of petals	pink	white
Branching density	sparse	sparse
Stem pubescence	slightly hairy	hairy
Color of stem	dark green and red	green
Degree of lignification	ligneous	slightly ligneous
Foliage density	medium	dense
Shape of leaf blade	ovate	rhomboid
Leaf margin	denticulate	denticulate
Shape of leaf apex	acute	rounded

**Table 3:** Morphological traits of investigated plants cd.

	Common Oregano *O. vulgare* ssp. *vulgare*	Greek Oregano *O. vulgare* ssp. *hirtum*
Plant height (cm)	36.11 ± 1.93 *	26.15 ± 1.86
Number of shoots per plant	27.59 ± 2.32 *	22.77 ± 1.53
Number of internodes per shoot	8.44 ± 1.56	7.60 ± 0.95
Fresh weight of herb (g × plant^−1^)	63.81 ± 13.0	49.17 ± 13.55
Dry weight of herb (g × plant^−1^)	16.71 ± 2.73	13.28 ± 3.22

Values marked in rows with ‘*’ differ at *p* < 0.05.

**Table 4:** Leaves area and density of glandular trichomes (GT) on the leaves.

	Common Oregano *O. vulgare* ssp. *vulgare*	Greek Oregano *O. vulgare* ssp. *hirtum*
Leaf area (mm^2^)	78.27 ± 5.50 *	61.76 ± 5.84
Density of GT on adaxial surface of leaf (number per 1 mm^2^)	0.78 ± 0.05	4.78 ± 0.65 *
Density of GT on abaxial surface of leaf (number per 1 mm^2^)	1.17 ± 0.19	4.85 ± 0.59 *

Values marked in rows with ‘*’ differ at *p* < 0.05.
